# Preclinical toxicological assessment of a novel monoclonal antibody targeting human platelet-derived growth factor CC (PDGF-CC) in PDGF-CC^hum^ mice

**DOI:** 10.1371/journal.pone.0200649

**Published:** 2018-07-18

**Authors:** Manuel Zeitelhofer, Hong Li, Milena Z. Adzemovic, Ingrid Nilsson, Lars Muhl, Andrew M. Scott, Ulf Eriksson

**Affiliations:** 1 Department of Medical Biochemistry and Biophysics, Karolinska Institutet, Stockholm, Sweden; 2 Olivia Newton-John Cancer Research Institute, and School of Cancer Medicine, La Trobe University, Melbourne, Australia; Duke University School of Medicine, UNITED STATES

## Abstract

Platelet-derived growth factor CC (PDGF-CC) is important during foetal development but also in pathogenesis of neurologic diseases, cancer and fibrosis. We have previously demonstrated that blocking the PDGF-CC/PDGF receptor alpha (PDGFRα) axis resulted in reduction of stroke volume and cerebrovascular permeability after experimentally induced stroke. Recently, we could translate these findings into the clinic showing that imatinib, a small tyrosine kinase inhibitor targeting PDGF receptors, can significantly improve neurological outcome after ischemic stroke in human. Herein we report preclinical toxicological analyses of our newly generated monoclonal anti-human PDGF-CC antibody 6B3 (mAb 6B3) in PDGF-CC humanized mice. Beside histological organ assessment, we also analysed serum, urine, haematological parameters and the general health status of the treated mice. We could not find any indications that mAb 6B3 is toxic or has other significant side effects neither in short, nor in long treatment regimens. Our results indicate that mAb 6B3 can be further developed for clinical use. This opens up the possibility to assess the therapeutic potential of blocking PDGF-CC in diverse pathological conditions such as neurologic diseases, cancer and fibrosis.

## Introduction

Platelet-derived growth factor (PDGF)-CC is a member of the PDGF family and consists of a growth factor (GFD) and a N-terminal CUB (homology to complement components C1r/C1s, Uegf, BMP1) domain [1]. The CUB domain sterically blocks the receptor binding surface in the GFD and has to be proteolytically removed in order to release the GFD dimer and subsequently allow PDGF-CC to bind its receptor, PDGFRα [2]. Tissue type plasminogen activator (tPA) was identified as an enzyme that cleaves latent PDGF-CC [3]. We and others have previously shown that injection of tPA or active PDGF-CC into the cerebral spinal fluid (CSF) leads to increased permeability of the blood brain barrier (BBB) [4],[5], a hallmark of diverse pathological conditions of the central nervous system (CNS) [6]. Notably, the thrombolytic agent tPA is the only pharmaceutical treatment for ischemic stroke that is currently approved by the US Food and Drug Administration (FDA). The treatment is, however, associated with neurotoxicity and an increased risk for haemorrhage, which affects 7% of stroke patients who received thrombolytic treatment and is associated with increased mortality [7]. The increased haemorrhage risk is possibly due to tPA mediated PDGF-CC activation which has been shown to increase BBB leakage [5]. Moreover, tPA can be administrated only within the first 4.5 hours after onset of the ischemic insult to achieve optimal therapeutic results [8]. Notably, up to 90% of all patients affected by stroke do not arrive in time to a hospital in order to receive tPA treatment [9]. Hence, blocking the side effects of tPA therapy would be of outmost importance to enable the treatment to a broader population of stroke patients by increasing the therapeutic window of tPA therapy.

We have shown that blocking PDGF receptor signalling with the small molecule tyrosine kinase inhibitor imatinib can reduce stroke volume in middle cerebral artery occlusion (MCAO), a mouse model of ischemic cerebrovascular insult [5]. Moreover, imatinib reduced BBB leakage and haemorrhagic complications when tPA was applied five hours after onset of MCAO [5]. In light of these experimental data, we recently performed a phase II randomized trial with imatinib in patients with acute ischemic stroke treated with intravenous thrombolysis [10]. This study showed that imatinib significantly ameliorates neurological symptoms with an improvement of 0.6 points per 100 mg imatinib on the National Institutes of Health Stroke Scale (NIHSS). In the high dose group that received 800 mg imatinib, the mean adjusted NIHSS improvements were 5 points in comparison to the patients treated with tPA only. In addition, inhibition of the PDGF-CC/PDGFRα axis has been shown to reduce BBB dysfunction, and has beneficial therapeutic effects in animal models of spinal cord injury (SCI) [11], multiple sclerosis (MS) [12], traumatic brain injury (TBI) [13], seizures [14] and amyotrophic lateral sclerosis (ALS) [15].

Apart from neurological diseases involving BBB disruption, it has been shown that PDGF-CC can facilitate tumour growth by recruiting cancer-associated fibroblasts (CAFs) and by increasing tumour angiogenesis [16]. Interestingly, PDGF-CC mediated angiogenic and tumorigenic properties of fibroblasts were observed in tumours refractory to anti-VEGFA treatment [17]. Further, siRNA-mediated reduction of PDGF-CC expression reversed resistance to cisplatin and thus improved the outcome in models of squamous cell carcinoma [18]. It has also been reported that overexpression of PDGF-CC *in vivo* resulted in liver fibrosis, steatosis and hepatocellular carcinoma [19] and that PDGF-CC mediates renal interstitial fibrosis [20]. Heart specific overexpression of PDGF-CC resulted in cardiac fibrosis, hypertrophy and dilated cardiomyopathy [21]. We have previously shown that *Pdgfc* deficiency in C57BL/6 mice is associated with various congenital defects [22] which were also observed in *tPA* deficient mice [23]. In addition it has been demonstrated that *Pdgfc* deficient mice with 129/Sv background die in the perinatal period due to feeding and respiratory difficulties associated with a complete cleft of the secondary palate [24]. Thus, *Pdgfc* deficient mice are not suitable for analysing the potential therapeutic effect of PDGF-CC to treat numerous neurological disorders, cancer and fibrosis.

Thus, we aimed to generate a transgenic mouse expressing PDGF-CC with a humanized GFD (PDGF-CC^hum^) that would allow us to neutralize PDGF-CC signalling *in vivo* using the anti-human PDGF-CC antibody 6B3 (mAb 6B3). Here we report the generation and characterisation of PDGF-CC^hum^ mice, and evaluated the toxological effects of neutralising PDGF-CC antibody 6B3 in these mice.

## Material and methods

### Generation of PDGF-CC^hum^ mice

The C57BL/6NTac-Pdgfc ^tm3633(K242T, K246R, R299S, K318R, N342S, A343T)Arte^ mice were generated by Taconic (Cologne, Germany) and are referred to as PDGF-CC^hum^ throughout the publication. The targeting strategy was based on NCBI transcript NM_019971_2. Exon 1 contains the translation initiation codon. To exchange the mouse with the human *PDGFC* six mutations were introduced into the mouse *Pdgfc* sequence. K242T, K246R and R299S mutations have been introduced into exon 5. The K318R, N342S and A343T mutations have been introduced into exon 6. These mutations correspond to the human amino acid sequence. Mouse genomic fragments (obtained from the C57BL/6J RPCIB-731 BAC library) and selected features (such as recombination sites and selection markers) were assembled into a targeting vector. If necessary, additional fragments were amplified by qPCR and subcloned. Positive selection markers have been flanked by FRT (Neomycin resistance—NeoR) and F3 (Puromycin resistance- PuroR) sites and have been inserted into intron 4 and intron 5, respectively. The targeting vector has been generated using BAC clones from the C57BL/6J RPCIB-731 BAC library and was transfected into the TaconicArtemis C57BL/6N Tac embryonic stem cell (ES) line. Homologous recombinant clones were isolated using double positive (NeoR and PuroR) and negative (Thymidine kinase—Tk) selections. The constitutive humanized allele was obtained after *in vitro* Flp-mediated removal of the selection marker. This allele expressed the mutated PDGF-CC K242T, K246R, R299S, K318R, N342S, A343T protein. The remaining recombination sites were located in non-conserved regions of the genome. PDGF-CC^hum^ mice are available upon request.

### Ethical statement

All experiments in this study were approved and performed in accordance with the guidelines from the Swedish National Board for Laboratory Animals and the European Community Council Directive (86/609/EEC) under the ethical permit N249/15, which was approved by the North Stockholm Animal Ethics Committee.

### Animals and antibody treatment

PDGF-CC^hum^ were originally obtained from Taconic (Germany). The generation of *Pdgfc*–deficient mice has been described previously [24]. Animals were housed in the Scheele animal facility at the Karolinska Institute (Stockholm, Sweden) in a pathogen-free and climate-controlled environment in polystyrene cages containing aspen wood shavings with free access to standard rodent chow and water with regulated 12-hour light/dark cycles.

Four week old male or female PDGF-CC^hum^ mice were injected intraperitoneally twice a week for either 10 days, 4 or 15 weeks with a weekly dose of 30 mg/kg (15 mg/kg per treatment) of either anti-PDGF-CC (6B3) or a control antibody (BM4).

### Cage side observations, weight measurements and urine analysis

Animals were monitored twice weekly according to the KI assessment checklist of health [25]. Thus the general health condition, porphyrin production, eyes, movements and posture, piloerection, respiration and skin were monitored. Once per week the animals were weighed. Two days before sacrificing urine samples were analysed using multistix (Siemens) or stored at -80 for further analysis. The mouse organs (n = 5 per treatment) were weighed upon harvesting using an analytical scale (Sartorius).

### Histopathological analyses and immunofluorescence (IF)

Animals were anesthetized using 4% isoflurane and perfused with HBSS followed by 4% paraformaldehyde (PFA). After 4h long fixation in 4% PFA, the organs were cryo-protected in 20% sucrose, 4°C over night (o/n) or paraffin embedded. Twelve μm thick cryosections were used for IF as following: after air-drying for 30 minutes, 10 minutes long permeabilization step was performed in PBS/0,2% Triton-x100. Blocking of unspecific binding was performed using PBS/10%FBS (blocking solution). Subsequently, primary antibodies (humanized anti-human mAb 6B3 detecting human PDGF-CC and neuronal marker anti-NeuN (Merck, MAB377) were diluted in the blocking solution and applied o/n at 4°C. The signal was visualized using Alexa-Fluor conjugated secondary antibodies (Invitrogen). DAPI was used for nucleus staining. IF images were captured using a confocal microscope (Zeiss LSM700) and shown as 2D renderings of 10 μm- thick z-stacks.

Organ morphology was assessed on 4 μm- tick paraffin sections stained with Hematoxylin & Eosin (H&E) according to a standard protocol [12]. Representative images were captured using inverted microscope Axio Observer Z1 (Zeiss, Germany).

### Clinical chemistry and hematology

Clinical chemistry and hematology analyses were performed by the University Animal Hospital, Swedish University of Agricultural Sciences, Uppsala, Sweden. Mouse serum (n = 5 per sex and treatment group, respectively) was harvested after 15 weeks of antibody treatment. ASAT, ALAT, albumin and calcium were analysed with spectrophotometry and the electrolytes (chloride, sodium and potassium) as well as conjugated bilirubin, total bilirubin, cholesterol, lipase, magnesium, triglycerides, amylase, iron, creatinine and creatine kinase were analysed with an ion specific electrode on an automated clinical chemistry analyser Architect c4000 (Abbott Laboratories, Abbott Park, IL, US) with commercial reagents from Abbott Laboratories. All whole blood samples were analysed by an automated haematology analyzer as well as manually, using a microscope. Leucocytes, neutrophils, eosinophils, basophiles, lymphocytes, monocytes, haemoglobin, erythrocytes, thrombocytes and reticulocytes were counted, mean cell haemoglobin concentration (MCHC) and mean cell volume (MCV) were calculated.

### mRNA generation and real time PCR analysis

mRNA was extracted from various organs using the RNeasy kit (Qiagen, Germany) and the QIAcube (Qiagen) including on column DNA-digestion for fully automated sample preparation. RNA concentration and purity was determined through measurement of A260/A280 ratios with a NanoDrop ND-1000 Spectrophotometer (NanoDrop Technologies, Wilmington, USA). Confirmation of RNA quality was assessed using the Agilent 2100 Bioanalyzer (Agilent Technologies, Santa Clara, CA, USA). mRNA was subsequently used for real time PCR analysis. cDNA was prepared using the iScript kit (Biorrad, Hercules, USA).

Real-Time quantitative PCR was performed using KAPA SYBR FAST qPCR Kit (Kapa Biosystems, KM4101) in Rotor-Gene Q Real-Time PCR thermal cycler (Qiagen) according to the manufacturers’ instructions. Expression levels were normalized to the expression of L19 (GGTGACCTGGATGAGAAGGA and TTCAAGCTTGTGGATGTGCTC). The following primers were used: Pdgfc^hum^: TCT CCT CAC CGA AGA GGT AAG GCT and GCT CTT CCC GTA TGG ACA CTG. mouse Pdgfc: TCT CCT CAA GGA AGA GGT AAA ACT and GCT CTT CCC GTA TGG ACA CTG, Pdgfra: TGG CAT GAT GGT CGA TTC TA and CGC TGA GGT GGT AGA AGG AG.

### Statistical analysis

GraphPad Prism software was used, and p values < 0.05 were considered to indicate statistical significance. Data are presented as mean ± SEM, and the number (n) of data points is from individual mice. For analyses of mouse data ordinary one-way ANOVA with Tukey’s test was used if more than two groups were compared, and Student’s unpaired t test analysis was used for two-group comparisons.

## Results

### Generation and characterization of the PDGF-CC^hum^ mouse strain

**Fig 1:**
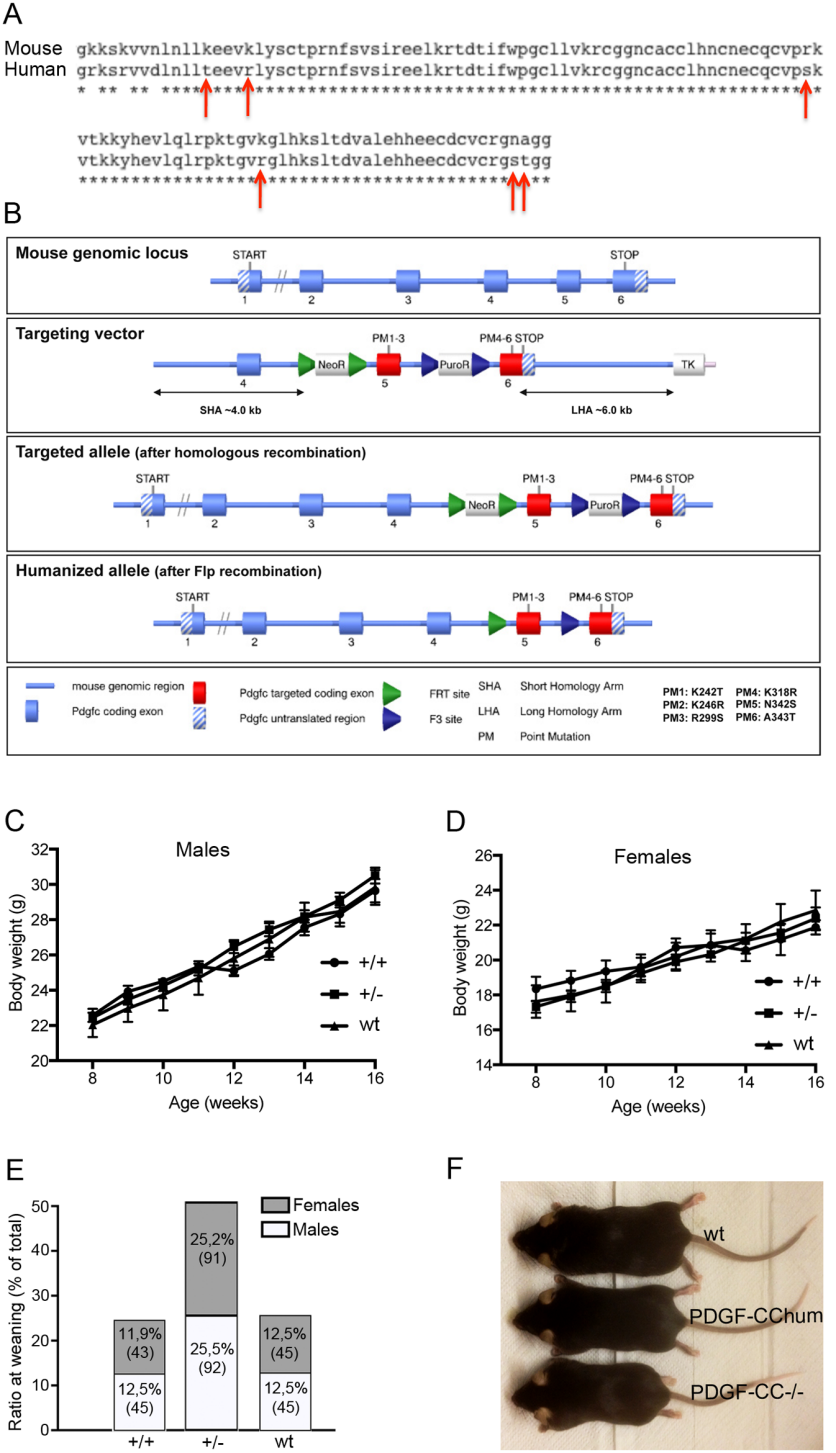
Construction and characterization of PDGF-CC^hum^ mice. The alignment of mouse and human PDGF-CC amino acid sequence is displayed. Red arrows indicate those amino acids that were subjected to point mutations (A). To exchange the mouse with the human *PDGFC* sequence 6 mutations were introduced into the mouse *Pdgfc* sequence. K242T, K246R and R299S mutations have been introduced into exon 5. The K318R, N342S and A343T mutations have been introduced into exon 6. Mouse genomic fragments and selected features (such as recombination sites and selection markers) were assembled into a targeting vector together with the 6 point mutations. Positive selection markers have been flanked by FRT (Neomycin resistance—NeoR) and F3 (Puromycin resistance- PuroR) sites and have been inserted into intron 4 and intron 5, respectively. The targeting vector was subsequently transfected into the TaconicArtemis C57BL/6N Tac embryonic stem cell (ES) line. The constitutive humanized allele was obtained after *in vitro* Flp-mediated removal of the selection marker. This allele expressed the mutated PDGF-CC K242T, K246R, R299S, K318R, N342S, A343T protein (B). Weight curve between 8 and 16 weeks of age in male (C) and female (D) PDGF-CC^hum^. Values in C-D represent mean +/- SEM (n = 10 for each genotype and gender) Distribution of genotypes and gender at weaning from heterozygous breedings (n = 57 litters). PDGF-CC^hum^ show Mendelian distribution of genotypes and even distribution of sexes (E). Comparison of the body size between PDGFCC^hum^, PDGF-CC^-/-^ and wt littermate (F).

We have generated and characterized a novel monoclonal antibody against human PDGF-CC called 6B3 [26]. mAb 6B3 was shown to block ligand induced phosphorylation of PDGFRα *in vitro* and blocks PDGF-CC mediated disruption of the blood retinal barrier (BRB) *in vivo*. Since the antibody specifically recognizes human PDGF-CC, we generated a transgenic mouse containing *Pdgfc* with a partially humanized GFD. Notably, mouse and human PDGF-CC GFD show high homology (Fig 1A). For humanization, 6 mutations were introduced into the GFD of the mouse *Pdgfc* genomic sequence. K242T, K246R and R299S mutations were introduced into exon 5 and the K318R, N342S and A343T mutations were introduced into exon 6 (Fig 1B). The constitutive humanized allele was obtained after *in vitro* Flp-mediated removal of the selection marker. This allele expressed the mutated PDGF-CC K242T, K246R, R299S, K318R, N342S, A343T protein (Fig 1B).

**Fig 2:**
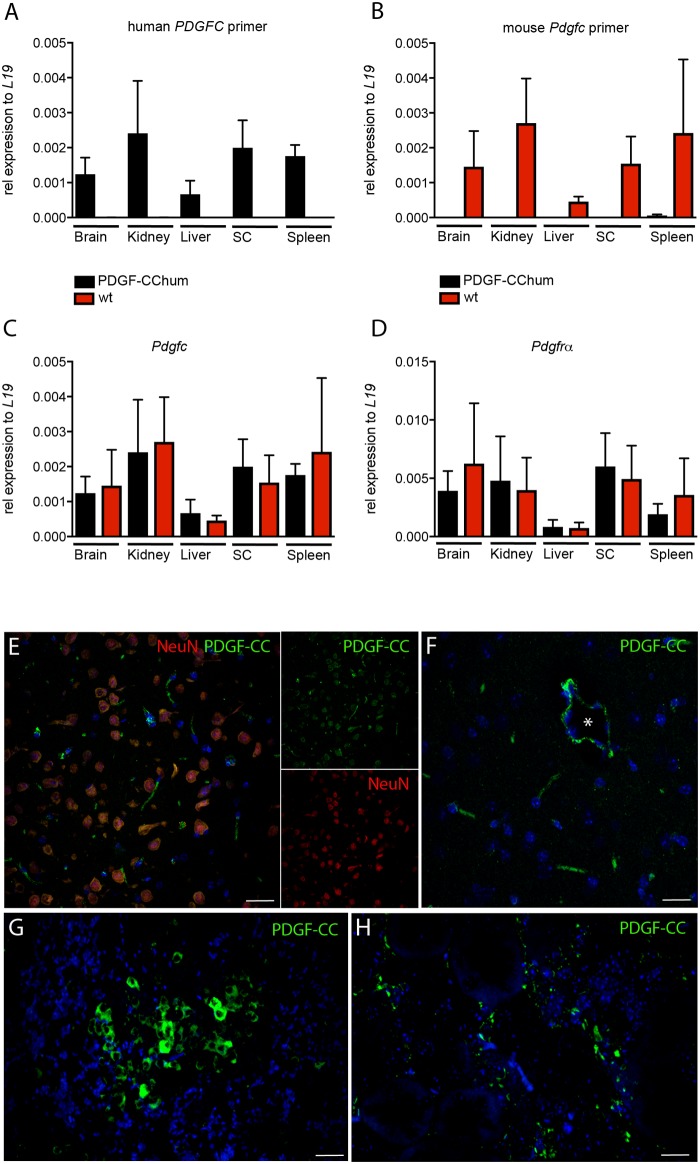
Humanized *Pdgfc* transcripts and PDGF-CC protein are expressed in PDGF-CC^hum^ mice. Primers specific for the mutated *Pdgfc* detect *Pdgfc* in PDGF-CC^hum^ but not in wt mice (A). Primers specific for mouse *Pdgfc* detect *Pdgfc* in wt but not in PDGF-CC^hum^ mice (B). *Pdgfc* (C) and *Pdgfrα* (D) transcript levels are similar in PDGF-CC^hum^ and wt mice (n = 3 for PDGF-CC^hum^ and wt mice, respectively). Values in A-D represent mean +/- SEM. Immunostaining with mAB 6B3 in the PDGF-CC^hum^ mice (E-H). shows PDGF-CC expression in neurons (E) and blood vessels (E and F) in the brain. mAb 6B3 detects PDGF-CC in the spleen (G) and kidney (H). scale bars: 30 u μm (E-G) and 10 μm (H).

To determine whether the humanization process was successful we analysed the health status of the PDGF-CC^hum^ mice and the functionality of the partially humanized PDGF-CC protein. Both male and female PDGF-CC^hum^ mice had comparable body weight curve in comparison to their wild type (wt) littermates (Fig 1C and 1D). In addition we analysed the gender ratio at weaning. Heterozygous breeding demonstrated a normal Mendelian distribution of genotypes and genders (Fig 1E). As *Pdgfc*^*-/-*^ mice have reduced body weight [22] we compared PDGF-CC^hum^, *Pdgfc*^*-/-*^ and wt littermates. A representative image of the comparison of these 3 mouse strains showed that PGDF-CC^hum^ and wt littermate controls are larger than *Pdgfc*^*-/-*^ mice (Fig 1F). In addition, we confirmed the expression of *Pdgfc* mRNA with the partially humanized GFD (Fig 2A and 2B). Notably, mRNA levels of *Pdgfc* and *Pdgfrα* were similar in wt and PDGF-CC^hum^ mice (Fig 2C and 2D). Finally, we confirmed PDGF-CC protein expression in the PGDF-CC^hum^ mouse brain, spleen and kidney (Fig 2E–2H). PDGF-CC was expressed in cortical neurons (Fig 2E) and in blood vessels (Fig 2E and 2F) in the brain. In the spleen PDGF-CC was expressed in T cell rich zones (Fig 2G) and in the kidney in tubular structures (Fig 2H).

### Assessment of systemic toxicity of the anti-PDGF-CC antibody 6B3

**Fig 3:**
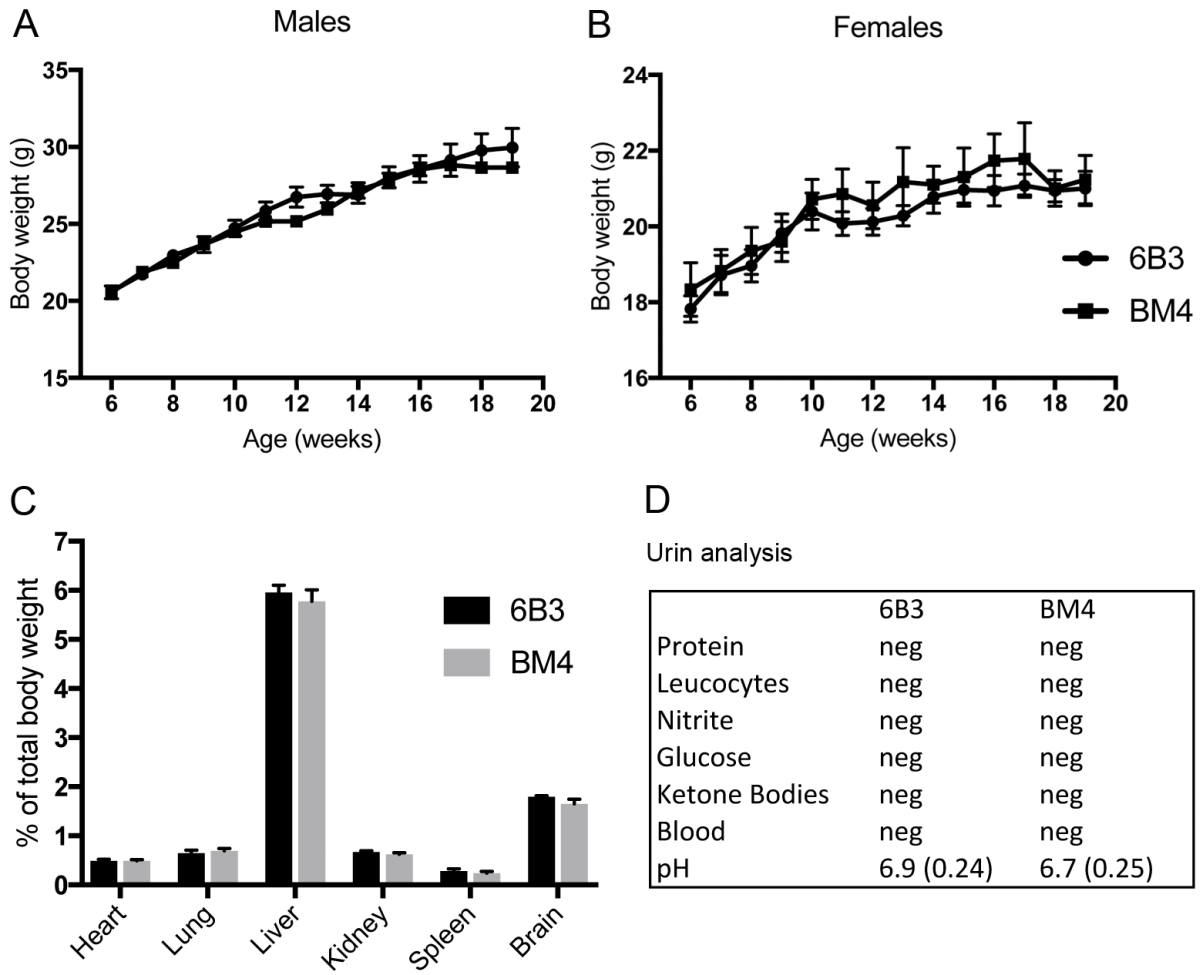
Toxicology assessment of 6B3. Female and male PDGFCC^hum^ were intraperitoneally injected weekly with 30 mg/kg 6B3 (n = 5 for each gender) or BM4 (n = 5 for each gender) for 15 weeks. Both male (A) and female (B) PDGF-CC^hum^ mice treated with 6B3 did not show any weight alterations compared to BM4 treated mice. Organs were harvested and the wet weight of the displayed organs did not differ between the 2 treatment groups (n = 5 for each treatment group) (C). Urine was harvested after 15 weeks and the parameters indicated in the table analyzed (n = 5 for each treatment group) For the ph value SEM is indicated in brackets. (D). Values in A-C represent mean +/- SEM.

To assess potential target toxicity of mAb 6B3, we injected PDGF-CC^hum^ mice intraperitoneally (i.p.) with a weekly dose of 30 mg/kg 6B3 or BM4, a control antibody. The mice were treated twice a week (15mg/kg per treatment) starting from 4 weeks of age for either 10 days, 4 or 15 weeks. Weight measurements were performed once a week until the end of the experiment. In contrast to *Pdgfc*^*-/-*^ mice that suffer from weight loss due to underdevelopment [22], we could not observe any difference in weight between BM4- and 6B3-treated male (Fig 3A) and female (Fig 3B) PDGF-CC^hum^ mice, respectively. Moreover, we could not detect any difference in weight of various organs upon treatment with 6B3 and BM4, respectively (Fig 3C). The general health status of the mice was assessed twice a week (S1 and S2 Tables). Both 6B3- and BM4-treated PDGF-CC^hum^ mice exhibited comparable activity, alertness and reaction to stimuli. Their movement and posture were also normal. The eyes were clear and showed no sign of infection/inflammation. We further observed a normal respiration rate as well as a regular bowel and urinary function in both groups. The urine analyses excluded abnormal levels of glucose or ketone bodies as well as the presence of leucocytes, nitrite and total protein (Fig 3D).

To further assess potential systemic side effects of the 6B3 treatment, we analysed several haematological parameters in PDGF-CC^hum^ mice after 10 days, and 15 weeks treatment with 6B3 and BM4, respectively. The analyses included parameters reflecting liver function such as alanine and aspartate transaminases (ALAT and ASAT), muscle condition (creatine kinase), renal function (creatinine), lipid metabolism, electrolyte status, iron concentration, albumin, bilirubin etc. Notably, all these values were within the standard range in both groups, excluding potential functional organ/system impairment (Table 1). We did not detect any difference in haemoglobin concentration, number of erythrocytes and various immune cells, between the groups after 10 days of treatment (S3 Table). However, a 15 week-long treatment with mAb 6B3 showed a slight difference in hematocrit (p = 0.049) and the number of red blood cells (p = 0.029) between the control (BM4) and treatment (6B3) group, although all values were still within the normal range (Table 2).

**Table 1:** Serum parameters after 15 weeks of 6B3 or BM4 injection.

**Parameter**	**Unit**	**Male Range 6B3**	**Mean 6B3**	**SEM 6B3**	**Male Range BM4**	**Mean BM4**	**SEM BM4**	**Reference value males**
ALAT	μkat/L	0.3–0.7	0.4	0.09	0.3–1.9	0.8	0.3	0.48–2.19
ASAT	μkat/L	1.3–3.7	2.3	0.5	2.3–9.0	5.5	1.2	0.76–6.51
Bilirubin. conjugated	μmol/L	1.8–2.0	1.8	0,0	1.8–1.8	1.8	0,0	
S-bilirubin. total	μmol/L	1.8–3.9	2.6	0.3	1.8–2.6	2,0	0.2	3.4–10.2
Calcium	mmol/L	3.0–3.2	3.1	0,0	2.9–3.3	3.1	0.1	2.43–3.13
Cholesterol	mmol/L	1.7–3.9	3.2	0.4	2.5–3.4	2.8	0.1	1.74–4.39
Lipase	μkat/L	1.2–20.0	5.1	3.7	1.3–4.9	2.1	0.7	
Magnesium	mmol/L	1.9–2.1	2,0	0,0	1.9–2.1	1.9	0.0	
Triglycerides	mmol/L	1.1–2.1	1.5	0.2	1.1–2.2	1.6	0.2	0.77–3.20
Hemolysis index		0.6–1.2	0.8	0.1	0.7–2.2	1.2	0.2	
Albumin	g/L	26.7–34.0	30.6	1.6	24.4–33.6	30.2	1.7	28–38
Amylas	μkat/L	17.0–93.0	36.3	14.3	22.4–49.2	28.4	5.2	
Iron	μmol/L	33.2–41.4	36.8	1.6	20.0–36.2	30.1	2.7	
kreatinin	μmol/L	15.1–18.0	16.6	0.5	11.6–16.0	14.8	0.8	17.39–43.48
Protein	g/L	53.1–65.3	60.2	2.4	53.2–64.6	59.6	1.9	48–70
CK	μkat/L	4.2–34.6	14.9	5.3	13.9–58.7	26.6	8.7	
Na	mmol/L	147.0–150.6	149.4	0.8	147.0–150.2	148.8	0.5	145.2–176.2
Cl	mmol/L	109.8–111.6	110.7	0.3	106.5–114.8	112.1	1.5	110.7–129.8
**Parameter**	**Unit**	**Female Range 6B3**	**Mean 6B3**	**SEM 6B3**	**Female Range BM4**	**Mean BM4**	**SEM BM4**	**Reference value females**
ALAT	μkat/L	0.3–0.4	0.3	0,0	0.3–5.4	1.4	1.0	0.46–3.32
ASAT	μkat/L	2.1–4.3	3,0	0.5	1.6–6.2	3.6	0.8	0.71–6.59
Bilirubin. conjugated	μmol/L	1.8–1.8	1.8	0,0	1.8–2.5	1.9	0.1	
S-bilirubin. total	μmol/L	2.3–3.1	2.6	0.2	1.8–4.7	2.9	0.5	3.4–10.2
Calcium	mmol/L	2.8–3.1	2.9	0.1	2.8–3.0	2.9	0.0	2.43–3.08
Cholesterol	mmol/L	2.2–2.3	2.3	0,0	1.8–2.1	2.0	0.0	1.43–4.26
Lipase	μkat/L	1.2–1.3	1.3	0,0	1.0–1.4	1.2	0.1	
Magnesium	mmol/L	1.8–1.9	1.8	0,0	1.8–1.9	1.8	0.0	
Triglycerides	mmol/L	1.0–1.4	1.2	0.1	0.8–1.4	1.1	0.1	0.86–3.32
Hemolysis index		0.8–1.0	0.9^a^	0.1	n.d.	n.d.	n.d.	
Albumin	g/L	33.3–34.9	34,0	0.3	31.6–34.4	32.9	0.5	24–43
Amylas	μkat/L	18.9–25.7	21.3	1.6	16.4–21.4	18.5	1.0	
Iron	μmol/L	32.5–38.4	35.5^a^	3,0	28.0–44.7	34.8	2.8	
kreatinin	μmol/L	11.6–15.8	14.3	0.9	12.0–17.7	15.1	1.2	17.39–43.48
Protein	g/L	59.6–62.3	60.4	0.6	54.1–57.2	56.7	0.9	48–72
CK	μkat/L	7.6–16.6	11.6	2.3	5.2–16.2	11.0	1.9	
Na	mmol/L		147.3^a^		146.9–149.7	148.6^b^	0.9	147.5–181.2
Cl	mmol/L		113.9^a^		113.7–117.7	115.6^b^	1.2	111.9–134.0

Female or male PDGF-CC^hum^ were i.p. injected with 6B3 (n = 5 for males and n = 4 for females unless stated otherwise) or BM4 (n = 5 for each gender unless stated otherwise) for 15 weeks and the serum parameters indicated in the table were analysed. The indicated reference values are from biochemistry analysis of C57BL/6 mice from Charles River. Reference values for conjugated bilirubin, lipase, magnesium amylase, iron, creatine kinase and hemolysis index were not available. All serum parameters were analysed with Student’s unpaired t test analysis was used and no significant difference was found for any parameter in males or females. a: The serum of only 1 female mouse in the 6B3 group could be tested for Na and Cl; b: The serum of 3 female mice in the BM4 group could be tested for Na and Cl.

**Table 2:** Hematology parameters after 15 weeks injections with 6B3 or BM4.

Parameter	Unit	Male Range 6B3	Mean 6B3	SEM 6B3	Male Range BM4	Mean BM4	SEM BM4	p value 6B3 vs BM4	Reference value*
**Red blood cells (RBC)**	10^6^/μL	9.0–9.4	9.24	0.07	8.3–9.2	8.8	0.15	0.029	9.48 or 10.62
**Hemoglobin (HGB)**	g/dL	13.0–13.2	13.1	0.05	12.3–13.3	12.8	0.20	0.163	14.2 or 16.0
**Hematocrit (HCT)**	% (of blood)	45–47	46.4	0.40	42–47	44.2	0.86	0.049	46.6 or 51.8
**Mean cell volume (MCV)**	fL	50–51	50.4	0.25	49–51	50.2	0.37	0.666	49.2 or 48.8
**Mean cell hemoglobin conc. (MCHC)**	g/dL	27.5–29.1	28.3	0.28	28.3–29.8	29.0	0.28	0.098	30.2 or 31.1
**Reticulocytes**	% (of RBC)	3.0–3.0	3.0	0	3.0–3.0	3.0	0		2.9
**White blood cells (WBC)**	10^3^/μL	2.2–7.6	4.38	0.15	1.0–4.2	2.34	0.53	0.145	8.9 or 3.1
**Neutrophiles (NEUT)**	10^3^/μL	0.3–5.1	2.02	1.05	0.1–0.3	0.18	0.04	0.117	1.44 or 1.17
**Eosinophiles (EOS)**	10^3^/μL	<0.1–0.2			<0.1–0.3				0.14 or 0.05
**Basophils (BASO)**	10^3^/μL	<0.1–<0.1			<0.1–<0.1				0.03 or 0.01
**Lymphocytes (LYMPH)**	10^3^/μL	1.7–2.5	2.06	0.15	0.9–3.6	1.96	0.46	0.842	6.87 or 1.8
**Monocytes (MONO)**	10^3^/μL	0.1–0.5	0.22	0.08	0.0–0.1	0.08	0.02	0.128	0.41 or 0.06

Male PDGFCC^hum^ were i.p. injected with 6B3 (n = 5) or BM4 (n = 5) for 15 weeks and hematologic parameters were analysed. All hematology parameters were analysed using Student’s unpaired t test and no significant difference was found for any parameter.

*Mean value of C57BL/6 mouse hematology from Jackson lab and Charles River, respectively. Reticulocyte reference value only available from Jackson lab

### Assessment of possible organ-specific toxicity of mAb 6B3

**Fig 4:**
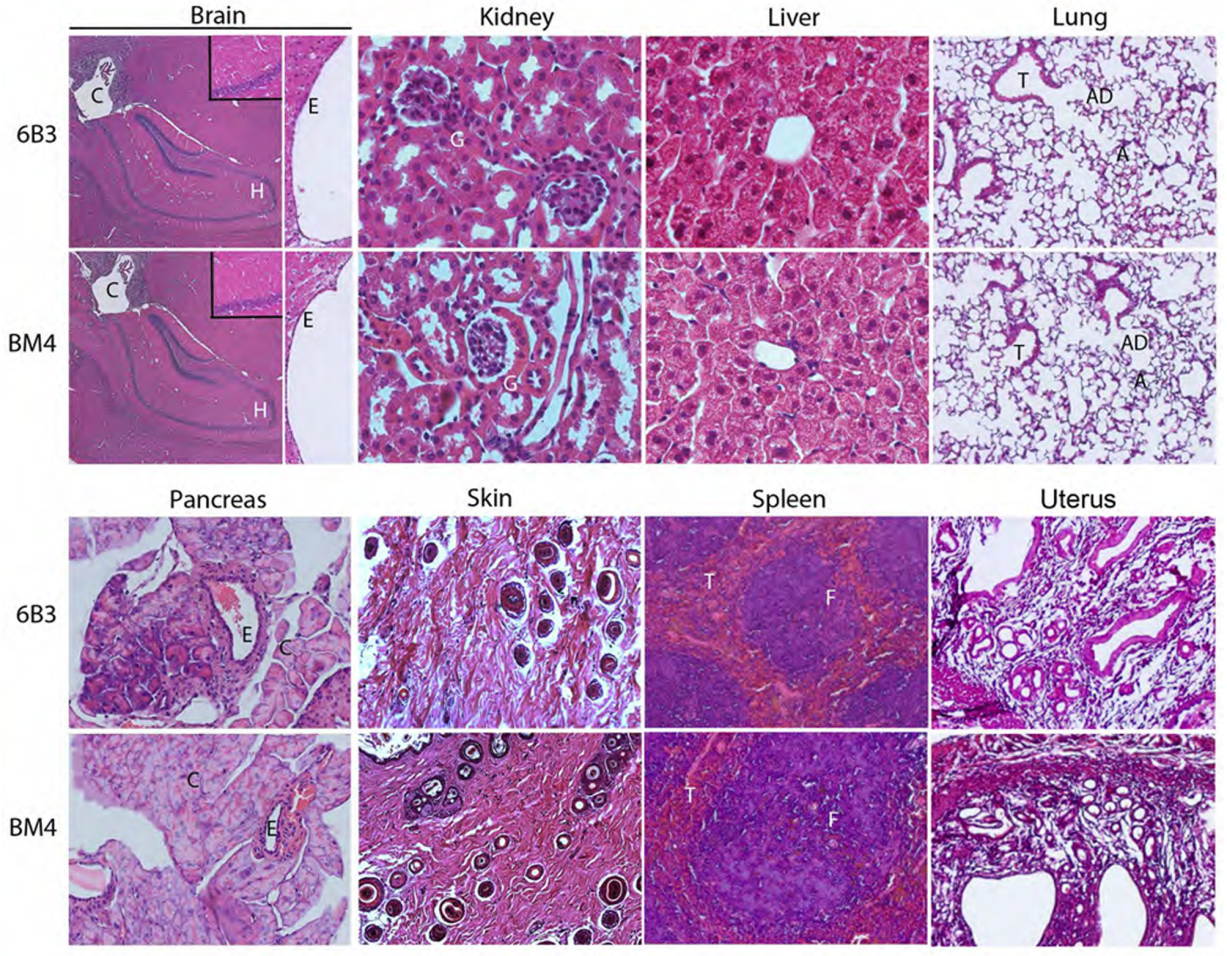
Long-term 6B3 treatment does not induce pathology of the major organs. Female and male PDGFCC^hum^ were intraperitoneally injected weekly with 30 mg/kg 6B3 (n = 5 for each gender) or BM4 (n = 5 for each gender) for 15 weeks. Tissue sections of brain (A), kidney (B), liver (C), lung (D), pancreas (E), skin (F), spleen (G) and uterus (H) were stained with HE and their morphology and pathology evaluated. Representative images of female mice are shown. H: Hippocampus, E: Ependymal cells; G: Glomerulus; RC:; T: Trachea, AD: Alveolar duct, A: Aveoli; C: Centroacinar cells, E: Exocrine duct; F: B cell-rich follicle, T: T cell-rich zone.

To further investigate potential side effects of a long-term treatment with mAb 6B3, we assessed various organs macroscopically and microscopically. 15 weeks after continuous treatment with 6B3 or BM4 antibodies, adrenal gland, brain, heart, kidney, liver, lung, pancreas, spleen, reproduction organs, skin, spinal cord, and thyroid gland in both groups did not exhibit signs of haemorrhage, edema, or any other macroscopic irregularity regarding organ morphology (Fig 4). In addition, we did not find any difference in weight of the brain, heart, kidney, liver, lung, and spleen between the treatment groups (Fig 3C). Histological analyses of the brain of 6B3 treated mice (Fig 4) excluded asymmetry of the lateral ventricles, hypoplasia of the septum and a distorted ependymal lining of the lateral ventricles commonly found in *Pdgfc*^*-/-*^ mice [22]. Tissue sections of the kidney cortex and medulla did not show any signs of inflammation or fibrosis, and glomeruli and surrounding tubular structure appeared normal (Fig 4). In addition, creatinine serum levels, an indicator of kidney disease, did not differ between the treatment groups (Table 1). The liver did not show any accumulation of lipids and the portal structure appeared normal with the portal vein in the center surrounded by the neat lining of sinusoids. In addition, hepatocytes did not show any signs of hyperplasia (Fig 4).

Deletion of *Pdgfc* at developmental stage on 129/Sv genetic background resulted in perinatal lethality in mice due to enlarged lungs with thickened mesenchyme and poorly differentiated cells [24],[27]. Notably, lungs of the 6B3 treated mice were not enlarged or with distorted alveoli (Fig 4). Further, *Pdgfc*^*-/-*^ mice displayed mild dermal mesenchymal hypoplasia and skin blistering on a 129/Sv background [24]. We could not detect any blister formation, rash or other skin pathologies upon macroscopical examination of the mouse skin. We therefore extended skin analyses to histology, which revealed no signs of abnormal hair follicles or dermal mesenchyme in the 6B3-treated mice (Fig 4). In addition 6B3-treated mice did not show any difference in morphology of the pancreas, spleen and uterus compared to BM4-treated mice (Fig 4).

## Discussion

We here present the phenotype of a novel PDGF-CC^hum^ mouse, and the results of preclinical toxicological assessment of our recently generated mAb 6B3 targeting PDGF-CC. The PDGF-CC^hum^ mice were phenotypically normal and fertile, allowing *in vivo* assessment of effects of neutralization of PDGF-CC with mAb 6B3. In addition to histological organ analyses, we analysed serum, urine, haematological parameters and the general health status of the treated mice. There was a slight difference in RBC and hematocrit between 6B3 and BM4 treated mice, however the parameters were within the normal range in both treatment groups, and thus unlikely to be of clinical relevance.

We have previously described in detail the binding affinity and functional activity of 6B3 [26] and shown that it blocks PDGF-CC induced phosphorylation of PDGFRα *in vitro* and thereby inhibits PDGFRα-mediated signalling. Moreover, the antibody could block PDGF-CC induced opening of the BRB after intraocular injection of active PDGF-CC, indicating its potential to block PDGFRα signalling also *in vivo* [26].

The partially humanized PDGF-CC mouse strain used in this study is a suitable model for generating a reliable toxicological assessment of mAb 6B3 due to a similar PDGF-CC tissue expression in mice and human [26],[28]. Moreover, the antibody has a high affinity to human PDGF-CC, implying a possibility for use of 6B3 in human therapy. Similar to our approach, humanized mice expressing human CD4 have been successfully used for toxicological assessment of a human anti-CD4 mAb keliximab, including pharmacokinetic/pharmacodynamic (PK/PD) profiling and single- and repeat-dose toxicity analyses [29],[30].

We have demonstrated that blocking PDGFRα signalling with the small molecule tyrosine kinase inhibitor imatinib decreased stroke volume and haemorrhagic transformation in MCAO [5]. Imatinib was also shown to improve neurological outcome in a phase II randomized trial in patients after acute ischemic stroke that were treated with intravenous thrombolysis [10]. In addition we demonstrated that PDGF-CC was upregulated in ALS and its animal model [15]. Hence, besides comparable expression pattern in mouse and human, similarities in the role of PDGF-CC in the human disease and the corresponding animal model additionally justify the use of the humanized mice for preclinical toxicological assessment instead of non-human primates.

Imatinib treatment was associated with cardiotoxicity in patients with chronic myeloid leukemia (CML), as well as with an abnormal bone and mineral metabolism [31]. In addition, imatinib is considered an unsuitable medication during pregnancy, based on preclinical data in mice [31, 32] and a retrospective study on pregnant women who received the treatment [33]. Furthermore, it has been observed that imatinib may alter liver parameters, and occasionally lead to liver failure [31]. Importantly, our results did not indicate any similar side effects upon treatment with mAb 6B3.

We have previously shown that *Pdgfc* deficiency in C57BL/6 mice is associated with various congenital defects such as abnormal vascular smooth muscle cell coverage, asymmetry of the lateral ventricles, hypoplasia of the septum and distorted ependymal lining of the lateral ventricles [22]. These defects were also observed in *tPA* deficient mice [23]. In addition, mice with *Pdgfc* deficiency and reduced *Pdgfrα* expression showed abnormal meningeal formation and neuronal over-migration in the cerebral cortex [27]. Notably, we could not find any of these defects in the 6B3-treated PDGF-CC^hum^ mice. *Pdgfc*^-/-^ mice also show decreased survival in the postnatal period (20%, instead of the 25% expected, survive when backcrossed onto a C57BL/6 background). In addition, spontaneous death was found after the postnatal period in *Pdgfc*^-/-^ mice. In contrast we could not observe any decrease in postnatal survival or spontaneous deaths after the postnatal period in PDGF-CC^hum^ mice. The oldest PDGF-CC^hum^ mice so far are 22–23 months old. They have been observed once a month since and we could not detect any alterations based on the clinical observations described in S1 and S2 Tables. In addition we could not detect weight loss in PDGF-CC^hum^ mice as seen in mice with *Pdgfc* ablation. Thus PDGF-CC^hum^ mice do not phenocopy *Pdgfc*^-/-^ mice and as a consequence the humanized PDGF-C in these mice is most probably cleaved and active. In the present study, we managed to address essential requirements for generating a reliable preclinical toxicological assessment [34] of mAb 6B3, which provided information about the status of organs/organ systems such as the liver, kidney, immune and hematopoietic system. Analyses of the tissue morphology did not show any differences in the 6B3 compared to the control group. The heart was excluded from histological analyses due to a very low expression level of PDGF-CC in this organ [21]. Nevertheless, we did not observe differences either in serum levels of creatine kinase or macroscopic changes in morphology and weight of the heart between the groups (Table 1, Fig 3). Thus, our clinical observations did not indicate any toxic effect in PDGF-CC^hum^ mice upon treatment with mAb 6B3 (S1 and S2 Tables).

We could not detect any significant difference between the groups regarding amount of the immune cells. These observations indicate that mAb 6B3 did not lead to any significant modulations of the immune system (Table 2 and S3 Table). Immune responses leading to the generation of anti-drug antibodies (ADA) can cause serious adverse events, but the most common problem is loss of efficacy, when ADAs bind the drug and neutralize its activity. Thus if ADAs against mAb 6B3 would be developed we most probably would have detected side effects in the hematological or organ analysis. In addition, we could not detect an increased number of serum lymphocytes of 6B3 compared to BM4 treated mice. We analyzed *Il2*, important for clonal T cell proliferation, and *Cd27* that plays a key role in regulating B-cell activation and immunoglobulin synthesis, in spleens after 3 months of 6B3 and BM4 injection and found similar levels than in non-injected controls (data not shown). If ADAs against mAb 6B3 would be developed we would expect activation and clonal expansion of both CD4+ T cells and B cells which then would differentiate into antibody producing plasma cells. Although being generally well tolerated, human recipients still may recognize parts of a therapeutic antibody as foreign, and thus activate immune and innate reactions [35]. Acute anaphylactic (IgE-mediated) or anaphylactoid reactions against a mAb in human cannot be ruled out entirely. A clinical manifestation may range from local skin reaction at the injection site, an influenza-like syndrome to potentially fatal acute anaphylaxis and systemic inflammatory response syndrome [36]. Notably, we could not observe any of these reactions in our experimental animals. Finally, it has been shown that mAbs may lead to tumour lysis syndrome (TlS) in patients [36]. To rule out this possible side effect, mAb 6B3 needs to be further tested in relevant mouse tumour model. Though we have performed a careful preclinical toxicology analysis we plan to perform a dose escalation experiment before taking mAb 6B3 into the clinics. In addition, we plan to perform toxicology and safety assessment together with PK/PD measurements in non-human primates.

Taken together, preclinical toxicological assessment of monoclonal anti- PDGF-CC mAb 6B3 did not indicate any significant systemic or organ alteration or side effects, upon both short- and long-term treatment with 6B3. It is planned to take 6B3 into the clinic with the ultimate aim of treating different neuropathologies, cancer or fibrosis.

## Supporting information

S1 Table Clinical observations (cage side) of PDGF-CC^hum^ i.p. injected with 6B3 or BM4 twice weekly for either 10 days, 4 or 15 weeks. Score 0.1 in the skin row indicates that the mice had small areas with fur loss. A score system from 0 to 0.4 was used. For explanation of the score system see S2 Table.
(DOCX)

S2 Table Explanation of the score system for the clinical observations. ^1^ Porphyrin = red-coloured secretion from eyes (or nose). ^2^ Unsteady and has difficulty coordinating movements. ^3^ Hairs stand up and coat appears harsh. Animal appears to be cold.
(DOCX)

S3 Table Hematology analysis after 10 days injection of 6B3 or BM4. Female PDGFCC^hum^ were i.p. injected with 6B3 (n = 5 for each gender) or BM4 (n = 5 for each gender) two times within 10 days and hematology was analysed. Statistics were calculated using Student’s unpaired t test. No significance was found for the analytes examined. Mean reference value of C57BL/6 mouse hematology are from Jackson lab and Charles River, respectively. Abbreviations: MCHC: mean cell hemoglobin concentration; MCV: mean cell volume.
(DOCX)
