# Supplementary material for: Preclinical toxicological assessment of a novel monoclonal antibody targeting human platelet-derived growth factor CC (PDGF-CC) in PDGF-CChum mice
Source: PLoS One. 2018 Jul 18;13(7):e0200649. doi: 10.1371/journal.pone.0200649 (PMC6051635; doi:10.1371/journal.pone.0200649)
Supplement: S1 Table — Score 0.1 in the skin row indicates that the mice had small areas with fur loss. A score system from 0 to 0.4 was used. For explanation of the score system see S2 Table. (DOCX) [file pone.0200649.s001.docx]

| **S1 Table.** | | | | | |
| --- | --- | --- | --- | --- | --- |
|  |  |  |  |  |  |
| \| **Observation** \| **6B3 males** \| **BM4 males** \| **6B3 females** \| **BM4 females** \| \| --- \| --- \| --- \| --- \| --- \| \| **General condition** \| 0 \| 0 \| 0 \| 0 \| \| **Porphyrin staining and/ or eye inflammation** \| 0 \| 0 \| 0 \| 0 \| \| **Movements and postures** \| 0 \| 0 \| 0 \| 0 \| \| **Piloerection** \| 0 \| 0 \| 0 \| 0 \| \| **Skin** \| 0.1 \| 0.1 \| 0.1 \| 0.1 \| \| **Weight** \| 0 \| 0 \| 0 \| 0 \| \| **Appetite** \| 0 \| 0 \| 0 \| 0 \| \| **Function** \| 0 \| 0 \| 0 \| 0 \| \| **Respiration** \| 0 \| 0 \| 0 \| 0 \|   **S1 Table.** Clinical observations (cage side) of PDGF-CC^hum^ i.p. injected with 6B3 or BM4 twice weekly for either 10 days, 4 or 15 weeks. Score 0.1 in the skin row indicates that the mice had small areas with fur loss. A score system from 0 to 0.4 was used. For explanation of the score system see S2 Table. | | | |  |  |
