# Supplementary material for: Preclinical toxicological assessment of a novel monoclonal antibody targeting human platelet-derived growth factor CC (PDGF-CC) in PDGF-CChum mice
Source: PLoS One. 2018 Jul 18;13(7):e0200649. doi: 10.1371/journal.pone.0200649 (PMC6051635; doi:10.1371/journal.pone.0200649)
Supplement: S2 Table — 1 Porphyrin = red-coloured secretion from eyes (or nose). 2 Unsteady and has difficulty coordinating movements. 3 Hairs stand up and coat appears harsh. Animal appears to be cold. (DOCX) [file pone.0200649.s002.docx]

**S2 Table.**

| **Observation** | **Score** | **Explanation** |
| --- | --- | --- |
| **General condition** | 0 | Awake, active, reacts to stimulation |
|  | 0.1 | Burrows in litter, hides, lies still but is startled when handled |
|  | 0.4 | Immobile, little or no voluntary movement. Burrows/hides. Presses head against cage bottom. Vocalizes. Extremely afraid and/or aggressive when handled |
| **Porphyrin staining ^1^ and or eye inflammation** | 0 | No discoloration, clean and clear eyes |
|  | 0.1 | Some porphyrin ^1^ and/or discharge around eyes and nose (can be difficult to see in coloured animals) |
|  | 0.4 | Obvious porphyrin ^1^ on ‘face’ and/or on legs and paws. Eye(s) closed, squints and/or discharge around eye(s) |
| **Movements and posture ^2^** | 0 | Normal |
|  | 0.1 | Moderate in-coordination ^2^ when animal is stimulated; hunched posture |
|  | 0.4 | Marked in-coordination ^2^, head held at angle, hunched posture and/or back, does not support itself on all four limbs and/or paralysis |
| **Piloerection ^3^** | 0 | Fur smooth and well-groomed |
|  | 0.1 | Moderate piloerection ^3^ |
|  | 0.4 | Severe piloerection ^3^, sticky and poorly groomed fur |
| **Skin** | 0 | Skin covered entirely with fur. No sores or other signs of injury |
|  | 0.1 | Small sores or scabs, no infection; scratching (signs of itching) |
|  | 0.4 | Bites or scratches itself or trauma from others. Signs of infection such as redness and/or pus or serious discharge; sticky and poorly groomed fur. Non-healing operation wounds or broken sutures |
| **Weight** | 0 | < 5% weight loss compared to weight "before experiment", day 1-3 |
|  | 0.1 | 5-10% weight loss compared to weight "before experiment", day 1-3 |
|  | 0.4 | 10-20 or >% weight loss compared to weight "before experiment", day 1-3 |
| **Appetite** | 0 | Normal appetite, eats dry food, food disappears from feed container or floor |
|  | 0.1 | No signs that animal has eaten dry food, drinks and exhibits no signs of dehydration |
|  | 0.4 | No interest in food or treats and/or appears dehydrated |
| **Function** | 0 | Normal bowel and urinary functions |
|  | 0.1 | Faeces looser than normal |
|  | 0.4 | Constipated or faeces are dry "raisin-like" or loose. No obvious urination or urine in larger amounts than normal and/or has strong odour |
| **Aspiration** | 0 | Normal respiration, not strained or wheezy |
|  | 0.4 | Breathes with open mouth, abdominal breathing or panting, crackle and/or gasping noises |

**S2 Table. Explanation of the score system for the clinical observations**

^1^ Porphyrin = red-coloured secretion from eyes (or nose).

^2^ Unsteady and has difficulty coordinating movements.

^3^ Hairs stand up and coat appears harsh. Animal appears to be cold.
