# Supplementary material for: Preclinical toxicological assessment of a novel monoclonal antibody targeting human platelet-derived growth factor CC (PDGF-CC) in PDGF-CChum mice
Source: PLoS One. 2018 Jul 18;13(7):e0200649. doi: 10.1371/journal.pone.0200649 (PMC6051635; doi:10.1371/journal.pone.0200649)
Supplement: S3 Table — Female PDGFCChum were i.p. injected with 6B3 (n = 5 for each gender) or BM4 (n = 5 for each gender) two times within 10 days and hematology was analysed. Statistics were calculated using Student’s unpaired t test. No significance was found for the analytes examined. Mean reference value of C57BL/6 mouse hematology are from Jackson lab and Charles River, respectively. Abbreviations: MCHC: mean cell hemoglobin concentration; MCV: mean cell volume. (DOCX) [file pone.0200649.s003.docx]

**S3 Table.**

| **Parameter** | **Unit** | **Female Range 6B3** | **Mean 6B3** | **SEM 6B3** | **Female Range BM4** | **Mean BM4** | **SEM BM4** | **Reference value*** |
| --- | --- | --- | --- | --- | --- | --- | --- | --- |
|  |  |  |  |  |  |  |  |  |
| **Red blood cells** | 10^6^/μL | 7.9 - 9.0 | 8.46 | 0.19 | 7.4 - 9.2 | 8.22 | 0.29 | 10.9 or 9.24 |
| **Hemoglobin** | g/dL | 12.1 - 14.0 | 13.14 | 0.32 | 11.5 - 14.0 | 12.7 | 3.98 | 16. 4 or 13.8 |
| **Hematocrit** | % (of blood) | 41 - 46 | 43.6 | 0.93 | 37 - 46 | 41.8 | 1.53 | 51. 2 or 45.4 |
| **MCV** | fL | 51- 53 | 51.8 | 0.37 | 50 - 52 | 51 | 0.45 | 47 - 49.2 |
| **MCHC** | g/dL | 29.4 - 30.6 | 30.12 | 0.24 | 29.5 - 30.7 | 30.34 | 0.22 | 32.1 or 30.7 |
| **Reticulocytes** | % (of RBC) | 3.0 - 4.0 | 3.6 | 0.24 | 1.96 - 4.0 | 3.2 | 0.2 | 3.1 |
| **White blood cells** | 10^3^/μL | 2.0 - 3.4 | 2.84 | 0.26 | 1.7 - 3.5 | 2.58 | 0.3 | 2.67 or 8.44 |
| **Neutrophiles** | 10^3^/μL | 0.2 - 0.2 | 0.2 | 0 | 0.2 - 0.4 | 0.26 | 0.04 | 0.23 or 1.19 |
| **Eosinophiles** | 10^3^/μL | <0.1 - 0.2 |  |  | <0.1 - <0.1 |  |  | 0.08 or 0.15 |
| **Basophils** | 10^3^/μL | <0.1 - <0.1 |  |  | <0.1 - <0.1 |  |  | 0.00 or 0.03 |
| **Lymphocytes** | 10^3^/μL | 1.7 - 3.0 | 2.44 | 0.24 | 1.4 - 3.0 | 2.1 | 0.26 | 2.32 or 6.71 |
| **Monocytes** | 10^3^/μL | 0.1 - 0.1 | 0.1 | 0 | 0.1 - 0.1 | 0.1 | 0 | 0.03 or 0.36 |

**S3 Table.** **Hematology analysis after 10 days injection of 6B3 or BM4.** Female PDGFCC^hum^ were i.p. injected with 6B3 (n=5 for each gender) or BM4 (n=5 for each gender) two times within 10 days and hematology was analysed. Statistics were calculated using Student’s unpaired t test. No significant differences between 6B3 and BM4-treated mice were found for the analytes examined. Mean reference value of C57BL/6 mouse hematology are from Jackson lab and Charles River, respectively. Abbreviations: MCHC: mean cell hemoglobin concentration; MCV: mean cell volume
